# Epigenetic effects of folate and related B vitamins on brain health throughout life: Scientific substantiation and translation of the evidence for health improvement strategies

**DOI:** 10.1111/nbu.12611

**Published:** 2023-02-21

**Authors:** A. Caffrey, Y. Lamers, M. M. Murphy, N. Letourneau, R. E. Irwin, K. Pentieva, M. Ward, A. Tan, A. Rojas‐Gómez, L. A. Santos‐Calderón, J. Canals‐Sans, B. M. Y. Leung, R. Bell, G. F. Giesbrecht, D. Dewey, C. J. Field, M. Kobor, C. P. Walsh, H. McNulty

**Affiliations:** ^1^ Nutrition Innovation Centre for Food and Health (NICHE), School of Biomedical Sciences Ulster University Coleraine UK; ^2^ British Columbia Children's Hospital Research Institute, Food Nutrition and Health Program, Faculty of Land and Food Systems The University of British Columbia Vancouver British Columbia Canada; ^3^ Unit of Preventive Medicine & Public Health, Department of Basic Medical Sciences, Faculty of Medicine & Health Sciences Universitat Rovira i Virgili, IISPV Reus Spain; ^4^ CIBEROBN, ISCIII Madrid Spain; ^5^ Faculty of Nursing and Cumming School of Medicine University of Calgary Calgary Alberta Canada; ^6^ Genomic Medicine Group, School of Biomedical Sciences Ulster University Coleraine UK; ^7^ Department of Psychology, Faculty of Educational Sciences and Psychology Universitat Rovira i Virgili Tarragona Spain; ^8^ Faulty of Health Sciences University of Lethbridge Lethbridge Alberta Canada; ^9^ Faculty of Agricultural, Life and Environment Science University of Alberta Edmonton Alberta Canada

**Keywords:** ageing, B vitamins, cognition, DNA methylation, folate, one‐carbon metabolism

## Abstract

Suboptimal status of folate and/or interrelated B vitamins (B_12_, B_6_ and riboflavin) can perturb one‐carbon metabolism and adversely affect brain development in early life and brain function in later life. Human studies show that maternal folate status during pregnancy is associated with cognitive development in the child, whilst optimal B vitamin status may help to prevent cognitive dysfunction in later life. The biological mechanisms explaining these relationships are not clear but may involve folate‐related DNA methylation of epigenetically controlled genes related to brain development and function. A better understanding of the mechanisms linking these B vitamins and the epigenome with brain health at critical stages of the lifecycle is necessary to support evidence‐based health improvement strategies. The *EpiBrain* project, a transnational collaboration involving partners in the United Kingdom, Canada and Spain, is investigating the nutrition–epigenome–brain relationship, particularly focussing on folate‐related epigenetic effects in relation to brain health outcomes. We are conducting new epigenetics analysis on bio‐banked samples from existing well‐characterised cohorts and randomised trials conducted in pregnancy and later life. Dietary, nutrient biomarker and epigenetic data will be linked with brain outcomes in children and older adults. In addition, we will investigate the nutrition–epigenome–brain relationship in B vitamin intervention trial participants using magnetoencephalography, a state‐of‐the‐art neuroimaging modality to assess neuronal functioning. The project outcomes will provide an improved understanding of the role of folate and related B vitamins in brain health, and the epigenetic mechanisms involved. The results are expected to provide scientific substantiation to support nutritional strategies for better brain health across the lifecycle.

## INTRODUCTION

Nutrition plays a fundamental role in the development, function and health of the human brain across the lifecycle. Optimal neurodevelopment requires a sufficiency and balance of macronutrients and key micronutrients including B vitamins in the periconceptional, perinatal and postpartum periods (Georgieff, 2007). It is well established from randomised controlled trials (RCTs) that periconceptional folic acid supplementation in mothers is effective in preventing neural tube defects (NTDs) in their babies (Czeizel & Dudás, 1992; MRC, 1991), evidence that has led to clear recommendations that are in place worldwide. To prevent NTDs, women are recommended to take a folic acid supplement (0.4 mg/day) from preconception until the end of the first trimester (WHO, 2017). Apart from shaping organ development in early pregnancy, nutrition in the first 1000 days of life may set the stage for cognitive function and brain health of the offspring into adulthood (Whalley et al., 2006). In later adult life, there is emerging evidence implicating deficiencies of certain nutrients in contributing to a greater risk of cognitive decline and demonstrating that a better nutritional status may be important in preserving cognitive health (Moore et al., 2018a). In particular, folate and the metabolically related B vitamins (B_12_, B_6_ and riboflavin) appear to be important for cognitive functioning in ageing (McNulty et al., 2019b). Notably, randomised trial evidence shows that B vitamin supplementation in older adults reduced brain atrophy of regions vulnerable to Alzheimer's disease by 7‐fold (Douaud et al., 2013). The biological mechanisms explaining these relationships are not clear but may involve folate‐related DNA methylation of epigenetically controlled genes related to brain development and function.

In the *EpiBrain* project, our aim is to investigate the nutrition–epigenome–brain relationship across the lifespan, focusing on folate and metabolically related B vitamins and their related epigenetic effects in relation to brain outcomes. We will use intervention and longitudinal studies across our three countries – Canada, the United Kingdom and Spain – where populations are exposed to different food fortification policies and supplementation practices, to ultimately provide scientific substantiation to support the development of effective health improvement strategies. This article provides a short overview of the background knowledge, the project objectives and the novel approaches that will be used to deliver the project outcomes.

## FUNCTIONAL AND BIOLOGICAL ROLES OF FOLATE AND THE RELATED B VITAMINS

**FIGURE 1:**
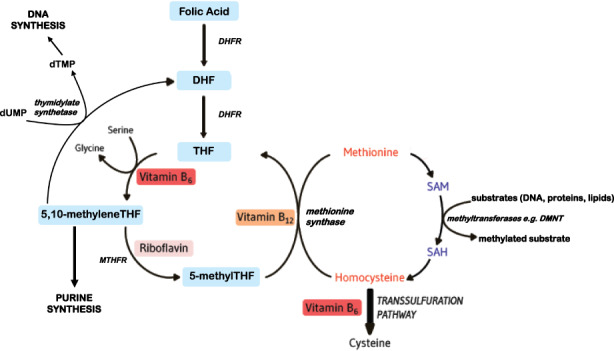
Overview of folate and related B vitamins in one‐carbon metabolism. DHF, dihydrofolate; DHFR, dihydrofolate reductase; DMNT, DNA methyltransferase; dTMP, deoxythymidine monophosphate; dUMP, deoxyuridine monophosphate; MTHFR, methylenetetrahydrofolate reductase; SAH, S‐adenosylhomocysteine; SAM, S‐adenosylmethionine; THF, tetrahydrofolate.

Folate plays an essential role in one‐carbon metabolism where it acts as a cofactor in DNA synthesis and repair, methylation processes and amino acid reactions (Figure 1). Within this network, folate in its various cofactor forms functions in mediating the transfer and utilisation of one‐carbon units (e.g., a methyl, formyl or formimino group) in metabolic pathways requiring close interaction with vitamin B_12_, vitamin B_6_ and riboflavin (Bailey et al., 2015). Reduced folates enter the one‐carbon cycle as tetrahydrofolate (THF) which acquires a carbon unit from serine in a vitamin B_6_‐dependent reaction to form 5,10‐methylene THF required for the synthesis of nucleic acids or is converted to 5‐methyl THF. Methylenetetrahydrofolate reductase (MTHFR) is the riboflavin (FAD)‐dependent enzyme that catalyses the reduction of 5,10‐methylene THF to 5‐methyl THF. Once formed, 5‐methyl THF is required for the remethylation of homocysteine to methionine via the vitamin B_12_‐dependent enzyme methionine synthase. Methionine, in turn, is required for the generation of S‐adenosylmethionine (SAM), the essential methyl donor for numerous methylation reactions including those required for the nervous system (Bailey et al., 2015).

Notably, this pathway is essential for the methylation of DNA, by DNA methyltransferases using SAM as a cofactor, which can play a key role in controlling gene expression in a process referred to as epigenetics (Armstrong, 2014). Epigenetics refers to changes in gene expression which occur without altering the underlying DNA sequence, often via histone modification, RNA interference or DNA methylation (Armstrong, 2014). DNA methylation is the most widely studied epigenetic mechanism for gene regulation and is dependent on the sufficient supply of methyl donors provided by folate and the metabolically related B vitamins via the formation of SAM within one‐carbon metabolism (Irwin et al., 2016).

## B VITAMINS AND BRAIN HEALTH ACROSS THE LIFESPAN

### Early life/pregnancy

The effect of maternal folate during pregnancy on cognitive development of the offspring has been investigated in several studies. Positive associations between self‐reported folic acid supplementation in pregnancy and children's cognitive development (including abilities, function and performance) have been shown (Julvez et al., 2009; Roth et al., 2011; Villamor et al., 2012). These findings are in general agreement with evidence that found reduced cognitive development in the offspring of mothers with suboptimal folate status (Schlotz et al., 2010). A systematic review (*n* = 14) concluded that low maternal folate status during pregnancy was associated with poorer offspring cognitive development (Veena et al., 2016). However, the evidence is not entirely consistent with two longitudinal observational studies, finding no significant associations between blood folate status in later pregnancy and child cognitive performance (Tamura et al., 2005) or infant neurodevelopment (Wu et al., 2012).

The Spanish partner of this consortium reported that moderately elevated maternal plasma homocysteine (a biomarker of low status of folate and related methyl donor B vitamins) at 2–10 weeks preconception was inversely associated with neurodevelopmental outcomes in children aged 4 months and 6 years (Murphy et al., 2017). The UK partner, for the first time in a double‐blinded RCT (the *FASSTT* trial), investigated the effects of extended maternal folic acid supplementation (at the recommended dose of 400 μg/day) in trimesters 2 and 3 of pregnancy and thus beyond the periconceptional period recommended for preventing NTDs (McNulty et al., 2013). Subsequently, the children were followed up at 7 years for the assessment of cognitive ability, and those born to folic acid supplemented versus placebo mothers were found to perform better in various domains, with higher test scores for verbal intelligence quotient (IQ), performance IQ and general language, and in full‐scale IQ when compared with a nationally representative sample of 7‐year‐old British children (McNulty et al., 2019a). Further follow‐up of the children at 11 years of age showed that the positive effects on verbal comprehension remained, whilst neuronal responses to a language task, as objectively assessed using magnetoencephalography, suggested more efficient processing of language in children from folic acid‐supplemented mothers (Caffrey et al., 2021).

### Variations in maternal folate status across countries

To prevent NTDs, supplementation with folic acid (400 μg/day) is recommended globally from preconception until the end of the first trimester (WHO, 2017). In most European countries, including the United Kingdom and Spain, this recommendation is the sole official guideline as regards the use of prenatal supplements. In Canada, women are recommended to take a daily multivitamin supplement containing 400 μg/day folic acid for at least 2–3 months before conception, throughout pregnancy and for 4–6 weeks postpartum or for as long as breastfeeding continues (Wilson et al., 2015; Health Canada, 2009). The extended use of multivitamin–multimineral supplements is intended not only to prevent NTDs in the periconceptional period, but to allow women to meet their dietary iron requirements in the second and third trimesters of pregnancy, and to achieve sufficient nutrient levels throughout pregnancy and during lactation. However, in Canada, many prenatal supplements contain 800–1000 μg/day folic acid, which is more than 2‐fold higher than the recommended dosage. Thus, in the Canadian *Alberta Pregnancy Outcomes and Nutrition* (*APrON*) cohort, elevated red blood cell (RBC) folate concentrations (i.e., >1360 nmol/L; Pfeiffer et al., 2007) were found in 45% and 60% of women in early and late pregnancy, respectively (Fayyaz et al., 2014).

Concerns have been raised about potential adverse metabolic, physiologic and health consequences of high folic acid intake, which are generally linked with unmetabolised folic acid appearing in the circulation. Folic acid is the fully oxidised folate form found in supplements and fortified foods that requires reduction to biologically active, reduced folate forms after ingestion. Although after ingestion folic acid is readily reduced by dihydrofolate reductase and converted to the THF folate forms, this is a very slow and variable process (Bailey & Ayling, 2009), and thus exposure to high oral doses of folic acid can result in the appearance of unmetabolised folic acid which is not a normal constituent of plasma or other tissues and may have adverse metabolic effects. High‐dose folic acid intake is thus hypothesised to affect intracellular methionine regeneration and nucleotide synthesis by enzyme inhibition (Lyon et al., 2020). Potential adverse health consequences associated with high‐dose folic acid are proposed to include impaired foetal growth, an aggravating interaction with vitamin B_12_ deficiency, and an increased risk of childhood diseases. The Spanish partner in this consortium recently observed in the *Reus‐Tarragona Birth Cohort* (*RTBC*) study that low vitamin B_12_ status in early pregnancy combined with elevated versus normal folate status was associated with exacerbation of low vitamin B_12_ status and with lower mean cell volume as pregnancy progressed (Solé‐Navais et al., 2018). Furthermore, elevated plasma folate (≥ 30 nmol/L) was observed in 78% of the women taking >400 μg/day supplemental folic acid. Folate status decreased in mid‐pregnancy when folic acid supplementation was discontinued, and the role of betaine in homocysteine remethylation was then enhanced (Fernàndez‐Roig et al., 2013). This can be explained by the fact that betaine, which is derived from dietary choline, is a substrate for the enzyme betaine‐homocysteine methyltransferase and therefore acts as an additional methyl donor that serves in the remethylation of homocysteine to form methionine independently of folate and vitamin B_12_.

### Later life

Achieving optimal B vitamin intake in older populations through fortification or supplementation programmes may slow cognitive decline, maintain neuropsychiatric health and in turn help to preserve a better quality of life in ageing. One notable RCT showed that B vitamin supplementation in older adults with moderately elevated homocysteine concentrations led to a decrease in cerebral atrophy, thereby slowing cognitive decline (Douaud et al., 2013). However, the biological mechanisms explaining the nutrient–brain relationship in later life remain unclear. The UK partner investigated the effect of B vitamins on brain health in later life in the large‐scale *Trinity‐Ulster‐Department of Agriculture* (*TUDA*) ageing cohort study (5186 participants, aged >60 years). A greater risk of depression was observed for participants in the bottom 20% of biomarker values for relevant B vitamins, particularly folate, while regular consumption of B vitamin‐fortified foods was associated with a reduced risk of depression (Moore et al., 2019). To investigate a causative relationship, *TUDA* participants (*n* = 249) subsequently completed an RCT (*BrainHOP*) receiving daily B vitamin supplements (folic acid [400 μg], vitamin B_12_ [10 μg], vitamin B_6_ [10 mg] and riboflavin [10 mg]) or placebo over a 2‐year period. This intervention with B vitamins was found to protect against visuospatial cognitive decline and in a pilot study involving a subset of the *BrainHOP* cohort (*n* = 48) who were also examined using magnetoencephalography, with the results suggesting a protective effect of B vitamins on neuronal function (Moore et al., 2018b). Final results from the *BrainHOP* trial are anticipated for publication in 2023.

## FOLATE‐RELATED DNA METHYLATION OF EPIGENETICALLY CONTROLLED GENES RELATED TO THE BRAIN

Epigenetics is described as the molecular interface mediating gene–nutrient interactions during critical periods throughout the lifecycle (Mehler, 2008). DNA methylation, a key epigenetic mechanism, affects gene expression and the synthesis and methylation of foetal DNA strands during early pregnancy. DNA methylation patterns are established during embryonic development and genome‐wide remodelling of DNA methylation occurs in the later stages of neural development (Irwin et al., 2016). DNA methylation profiles laid down in utero affect not only early growth and development but also physiologic function and health in adult life. Folate‐mediated alterations in epigenetic marks of essential genes, such as those implicated in neural development, were recently identified as potentially affecting brain function and health throughout the lifespan (Caffrey et al., 2018). A balanced synergy of methyl donor nutrients, nutrient–nutrient and nutrient–gene interactions within the one‐carbon metabolic network is essential for methylation reactions. Folate, as the core nutrient in the one‐carbon metabolic network, can be a limiting factor in DNA methylation. As previously outlined, reduced folate forms are regenerated for methyl donor reactions in pathways that are also dependent on vitamin B_12_, vitamin B_6_ and riboflavin. Choline and betaine are additional methyl donor nutrients that serve in the remethylation of homocysteine to form methionine via betaine‐homocysteine methyltransferase, whereas folate and vitamin B_12_ are required as cofactors in the more typical reaction for generating methionine as catalysed by methionine synthase (Figure 1).

From the *FASSTT* trial, analysis of loci using pyro assays identified changes in methylation at perinatal growth factor (*IGF2*), brain‐derived neurotrophic factor (*BDNF*) as well as at *LINE‐1*, an interspersed repetitive sequence used as a proxy of genome‐wide changes (Caffrey et al., 2018). To further investigate the effects of folic acid supplementation in the *FASSTT* trial, in preliminary analysis of cord blood DNA, we used the EPIC methylation array (Illumina). Overall, the folic acid‐supplemented samples showed more loss than gain of methylation, though the numbers of sites showing differences in methylation large enough to verify in the lab (>5%) were small. Gene ontology analysis highlighted significant enrichment for genes involved in cognition with a low false discovery rate (FDR). The top‐ranked differentially methylated region (DMR) from the epigenome‐wide association studies was located ~2 kb upstream of *ZFP57*, which encodes a zinc finger protein known to regulate imprinted genes (Irwin et al., 2019). A pyroassay designed to match this region showed good concordance in cord blood samples, and we showed that folic acid drives a similar gain in methylation in maternal blood samples.

## THE EPIBRAIN PROJECT

**FIGURE 2:**
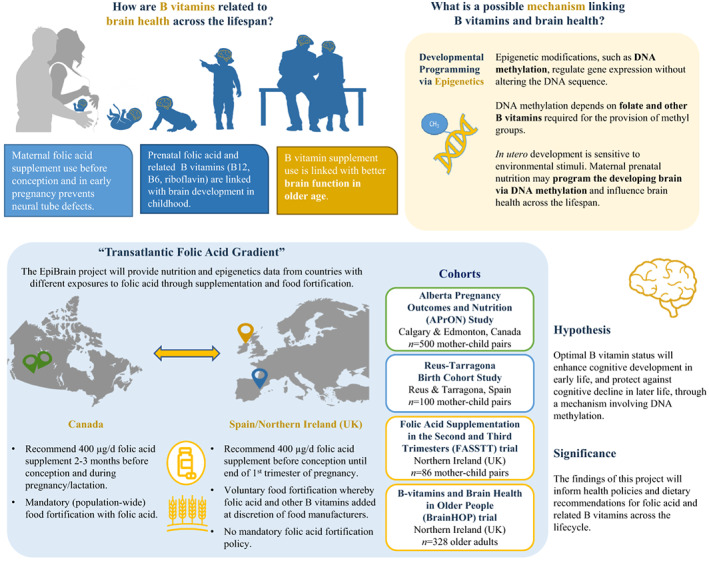
Graphical representation of the *EpiBrain* project: key objectives and related work package outcomes. The *EpiBrain* project is a secondary analysis of data from countries with different exposures to folic acid through supplementation and food fortification. Namely, *Alberta Pregnancy Outcomes and Nutrition* (*APrON*) Study (Kaplan et al., 2014), *Reus‐Tarragona Birth Cohort Study* (Fernàndez‐Roig et al., 2013), *Folic Acid Supplementation in the Second and Third Trimesters* (*FASSTT*) Trial (McNulty et al., 2013), and the *B vitamin and Brain Health in Older People* (*BrainHOP*) Trial (Moore et al., 2018b).

In the *Epigenetic effects of B vitamins on Brain health throughout life* (*EpiBrain*) project, our overarching aim is to improve understanding of the role of B vitamins in brain function in childhood and older age, and the related epigenetic mechanisms involved, with results expected to provide scientific substantiation to support nutritional strategies for sustaining better brain health throughout life. Specifically, we aim to investigate diet–epigenome relationships and their associations with child cognitive development focusing on early exposure to maternal B vitamins in utero and brain function (as assessed by magnetoencephalography) in early childhood. Considering the potential protective role of B vitamins against cognitive decline in later life, we are also investigating whether the diet–epigenetic relationship affecting cognitive development is related to cognition in later life. In 2018, the ERA–HDHL joint funding action under the “Nutrition & the Epigenome” call selected the *EpiBrain* project. The project is led by Prof Yvonne Lamers of the British Columbia Children's Hospital Research Institute and Food Nutrition and Health Program Faculty of Land and Food Systems, University of British Columbia (UBC), Canada, in collaboration with Prof Helene McNulty, Ulster University, Northern Ireland, United Kingdom and Prof Michelle Murphy, Universitat Rovira I Virgili, Spain. The *EpiBrain* project is a transnational interdisciplinary project that brings together research teams with expertise in nutrition, biochemistry, epigenetics, neurosciences, developmental psychology and clinical gerontology. A graphical representation of the *EpiBrain* project is shown in Figure 2.

The *EpiBrain* project will deliver epigenome‐wide association studies, and multivariate linear regression models will be completed using the Infinium methylation EPIC array that allows the interrogation of methylation patterns at the genome‐wide level, covering more than 450 k methylation sites. New EPIC array analysis of existing samples (from the *BrainHOP* trial and *RTBC* study) will be analysed at Dr Michael Kobor's laboratory at UBC, Canada. The EPIC and existing 450 k data from our cohort studies (as detailed below) can be cross‐compared using readily available packages.

## THE EPIBRAIN PROJECT COHORTS

The *EpiBrain* project offers a unique opportunity to investigate these associations in our populations – the United Kingdom, Canada and Spain – with very different exposures to folate and other methyl‐donor B vitamins, thus providing novel data to help advance our understanding of this important research area across the global range of dietary intakes and status of these nutrients.

### The Alberta Pregnancy Outcomes and Nutrition (APrON) study – From Canada

The *APrON* cohort study included pregnant women, their children and partners, with follow‐up visits at 6 years (Kaplan et al., 2014; Letourneau et al., 2022) and 12 years postpartum. Between 2009 and 2012, pregnant women (*n* = 2189) were included if they were >16 years at enrolment (up to 27 gestational weeks; GW), and living within Edmonton or Calgary, the two largest metropolitan areas in Alberta, Canada. Women were excluded if they were unable to answer questions in English or planned to move out of the study catchment prior to follow‐up at 3 months after delivery. The primary objectives of the *APrON* study were to determine the relationship of preconception, pregnancy and postpartum maternal nutrient intake and status with maternal mood, birth and obstetric outcomes, and child neurodevelopment. Women recruited at ≤GW13 were assessed once during each trimester, and women recruited at GW14–27 were assessed in the 2nd and 3rd trimesters only. As described in detail by Leung et al. (2015), comprehensive maternal nutrition, supplement use, biomarker, anthropometric (weight gain and fat gain) and mental health data were collected at multiple points in the pregnancy and the postpartum period, as well as obstetrical, birth and health outcomes of these pregnancies and neurodevelopmental outcomes of the children, including cognitive development. Sociodemographic information including education level, family income, family constellation, marital status, occupation, health status, obstetric history, medication use, smoking history, drugs and alcohol use was collected at the beginning of the study, and information was re‐checked at each study visit. For maternal dietary intake, pre‐pregnancy dietary intake in the year before pregnancy was assessed using a food frequency questionnaire (FFQ), specifically the Canadian Diet History Questionnaire Version 2 (Csizmadi et al., 2016). Maternal dietary intake in each trimester of pregnancy was assessed using a 24‐h recall and the multiple‐pass method in the form of in‐person interviews for the first 1000 participants and online 24‐h recalls thereafter (using Webspan, University of Waterloo, modified for pregnant women). Maternal blood samples were collected in each trimester for the preparation of serum, plasma, buffy coat and red blood cell (RBC) samples and are bio‐banked at −80°C. Fasting state was not required for the blood collection; food consumed in the 3 h prior to the venepuncture was recorded. Maternal biomarker analysis was completed for plasma and RBC folate, plasma holotranscobalamin (a direct indicator for vitamin B_12_ status) and plasma PLP (a direct indicator of vitamin B_6_ status) (Fayyaz et al., 2014).

Methylation analysis by 450 k has been carried out for children (*n* = 117 blood and 159 buccal samples) and is available as idat files or as cleaned datasets. Methylation analysis has also been carried out using the EPIC 850 K array (*n* = 217 blood and 283 buccal samples) and is available as idat files or as cleaned datasets. The child neurodevelopmental assessments at the ages 2, 3–4 and 5–7 years included a battery of broad‐based measures of cognitive and neuromotor development in combination with more fine‐grained assessments of specific skills (i.e., language, memory, executive function and motor function), following US National Children's Study recommendations. Cognition, language, social–emotional, motor and adaptive behaviour were assessed using the Bayley Scales of Infant Development Third Edition (Bayley‐III) at 2 years of age and the Wechsler Preschool and Primary Scales of Intelligence Fourth Edition (WPPSI‐IV) at 3–4 years and 5–7 years. The Bayley‐III and WPPSI‐IV have excellent reliability and validity. Further, parents completed standardised questionnaires of child behaviour and executive function at 2 years, 3–4 years and 5–7 years (i.e., Child Behaviour Checklist, Behaviour Assessment System for Children and Behaviour Rating Inventory of Executive Function‐Preschool).

### Planned analysis within the EpiBrain project

As part of the *EpiBrain* Project, we will measure maternal plasma concentrations of homocysteine, methionine, free choline, dimethylglycine, betaine and other one‐carbon metabolites, using isotope‐dilution liquid chromatography–tandem mass spectrometry (LC–MS/MS); the assay is set up and validated in Prof Yvonne Lamers' laboratory. For epigenetic analyses, maternal DNA was extracted from blood specimens using the Gentra Puregene Blood DNA purification kits on the Autopure LS Automated Nucleic Acid Purification Instrument; purified DNA samples are stored at −4°C (Kaplan et al., 2014). Either blood or buccal samples will be used for DNA extraction collected from the infant at 3 months of age (in ~5% of cases both were taken), allowing comparison of methylation signals in two tissues (maternal and infant). Advanced statistical models will be designed to analyse overarching and cohort‐specific research questions. We will design multivariate linear regression models to assess the maternal predictors of infant genome‐wide DNA methylation. Further, we will determine the association of maternal methyl donor nutrient intake and status before pregnancy and at different stages of pregnancy with neurodevelopmental outcomes.

### The Reus‐Tarragona birth cohort (RTBC) study – From Spain

The *RTBC* study is an ongoing prospective observational investigation of the association between prenatal nutritional status and outcomes at birth and at 7.5–8 years of age in the children. Extensive biological, clinical, lifestyle and socio‐economic data are collected from pregnant women from the 1st trimester (up to GW12) throughout pregnancy. Interview questions focused extensively on folic acid supplement use (brand, number of tablets and frequency of use in the year before, 2 months before, 1 month before and throughout pregnancy), at GW20 and in the last trimester. For maternal dietary intake data, women are asked to complete 2 FFQs, including specific questions about supplement use and breakfast cereal consumption, with the first FFQ at GW12 to cover the year leading up to pregnancy and the second FFQ after delivery to cover dietary intake during pregnancy. Blood samples are collected at multiple time points; maternal blood samples at <GW12, GW15, between GW24 and 27, GW34 and on admission to the labour ward with confirmed labour; cord blood samples are collected at delivery, paternal blood samples either during pregnancy or as near to the birth as possible, and child blood samples at 7.5 to 8 years of age.

Biomarker analysis in maternal and cord blood samples completed to date include haematological parameters, plasma and RBC folate, serum vitamin B_12_, total homocysteine, methionine, betaine, choline and related metabolites of the one‐carbon metabolic network. So far, the study has shown that low cobalamin and moderately elevated homocysteine concentrations in early pregnancy are associated with a metabolic score in mid‐childhood and specifically with fat mass index and insulin resistance in boys (Rojas‐Gómez et al., 2022). The child's neurodevelopmental assessments include a health check‐up with a complete study of adiposity, metabolic status and blood pressure. Cognitive and behavioural outcomes in the children are assessed using a battery of tests including the Neuropsychological Assessment of Executive Functions for Children (assesses maturity and cognitive performance in activities related to different components of executive functions for children aged 6–12 years and provides scores on Verbal Fluency, Planning; Interference and the Trail Making Test (TMT)), the School Neuropsychological Maturity Questionnaire (includes a neuropsychological evaluation of a broad repertoire of higher mental functions influencing the process of learning and behaviour in children aged 7–11 years), the CUMANES (to assess the children's visual and auditory memory) and the Wechsler Intelligence Scale for Children (WISC‐IV) to assess total IQ and several neuropsychological functions including Verbal Comprehension, Reasoning, Perception, Working Memory and Processing Speed.

### Planned analysis within the EpiBrain project

For the epigenetic analysis, genomic DNA will be extracted from maternal, cord and child bio‐banked leucocyte samples, and from placental tissue samples, and sent to the UBC for analysis using the EPIC array. We will then report on the associations between maternal status in methyl donor nutrients, B vitamins and DNA methylation patterns during pregnancy and their intergenerational association with DNA methylation in the cord. Similar models will be used to explore the association between maternal and paternal nutrient intake/status on neurodevelopment outcomes in the children. For the pilot studies, in fathers and in placenta samples collected from the RTBC cohort, similar models will be used to explore the association between paternal nutrient status and DNA methylation in the cord blood, placenta and child samples, as well as neurodevelopment in the child aged 7.5–8 years. Similarly, the associations between maternal nutrient status and DNA methylation in the placenta will be explored.

### The folic acid supplementation in the second and third trimesters (FASSTT) trial – From the United Kingdom

The *FASSTT* trial conducted in Northern Ireland aimed to investigate the effect of folic acid in pregnancy on the health outcomes of the mother and child (ISRCTN19917787). As described in detail elsewhere, healthy pregnant women aged 18–35 years, with singleton pregnancies, without pregnancy complications were recruited at GW14 from antenatal clinics in Northern Ireland between 2005 and 2006 (McNulty et al., 2013). Women included in the trial had taken folic acid supplements at the recommended dose (400 μg/days) during the 1st trimester of pregnancy; women were excluded from participation if they had not taken folic acid before or after conception or had taken folic acid at a dose >400 μg/days, were taking medications known to interfere with B vitamin metabolism, had undergone in vitro fertilisation treatment or had a previous NTD‐affected pregnancy. At the start of the 2nd trimester, eligible participants were randomly assigned to receive either 400 μg/day folic acid or a placebo until the end of the pregnancy. Dietary intake of the mothers during pregnancy and of the children at 7 and 11 years of age was assessed using a 4‐day food diary combined with an FFQ (Caffrey et al., 2021; McNulty et al., 2019a). Information on relevant health and lifestyle factors was also collected. Non‐fasting maternal blood samples were taken at GW14 (pre‐intervention) and GW36 (representative of post‐intervention), with corresponding cord blood samples collected at delivery. Blood samples from the 11‐year‐old children were collected during the follow‐up investigations (Caffrey et al., 2021). Maternal, cord and child blood biomarker analysis was completed for serum and RBC folate, plasma unmetabolised folic acid, serum vitamin B_12_, plasma PLP, erythrocyte glutathione reductase activation coefficient (EGRac; biomarker of riboflavin) and plasma homocysteine concentrations. Cognitive performance at the age of 7 years was assessed by the WPPSI‐III to test domains of Verbal, Performance, Processing Speed, General Language performance as well as Full Scale IQ. At the age of 11 years, the WISC‐IV (as described above) was used to assess cognitive outcomes. Functional brain activity was also assessed in a subset of the children at 11 years (*n* = 33) by magnetoencephalography, an imaging modality which passively measures the magnetic fields produced by neural activity and thus provides an objective evaluation of the nervous system maturity. For epigenetic analysis, pyrosequencing has already been used to analyse DNA methylation at nine candidate loci known to be regulated by methylation and showed significant differences at some genes (Caffrey et al., 2018). Genomic DNA was extracted from cord blood samples (*n* = 86) and analysed by EPIC array, which identified numerous DMR, including that at *ZFP57*. The latter was confirmed by pyroassay in a subset of maternal samples (n = 24) at GW14 and GW36 (Irwin et al., 2019).

### Planned analysis within the EpiBrain project

As part of the *EpiBrain* project, we are further analysing maternal samples by EPIC (we have existing EPIC data for 24 mothers and will add an additional 24 samples to give greater power to detect DMRs in the primary treated group). Recent analysis arising from the *EpiBrain* project shows that at the *CES1* gene, methylation changes at the promoters were important for regulating transcription. We also identified a second group which had a characteristic bimodal profile, with low promoter and high gene body methylation. In the latter, loss of methylation in the gene body is linked to decreases in transcription. This group included the *PRKAR1B*/*HEATR2* genes and the dopamine receptor regulator *PDE4C* (Ondičová et al., 2022). Loci showing the greatest differences in methylation will be validated by gene‐specific pyroassay. Further models will be designed to test whether parental diet, genes and lifestyle influence neurodevelopment in the offspring during infancy and childhood and whether epigenetic mechanisms are involved. Genomic DNA from the blood samples of children at 11 years of age will also be analysed by EPIC and linked with child neurodevelopmental assessments previously reported at ages 3 and 7 years (McNultyet al., 2019a) and 11 years (Caffrey et al., 2021).

### The B vitamins and brain health in older people (BrainHOP) trial – From the United Kingdom

The *BrainHOP* trial also conducted in Northern Ireland included adults aged 70 years and older who had previously participated in the *TUDA* study (ClinicalTrials.gov Identifier: NCT02664584). For the *TUDA* study, a total of 5186 community‐dwelling adults aged ≥60 years were recruited in 2008–2012 from centres in Northern Ireland (United Kingdom) and the Republic of Ireland. Of the 2093 participants recruited in Northern Ireland, 689 met the inclusion criteria for the *BrainHOP* trial (i.e., not a user of B vitamin supplements; Mini‐Mental State Examination [MMSE] score ≥21; a normal renal function defined as creatinine <130 μmol/L; and plasma homocysteine >12 μmol/L [suggestive of suboptimal status of one or more B vitamins]). The *BrainHOP* trial was conducted as a randomised double‐blinded 2‐year intervention trial. Of the 328 participants initially recruited (aged 78 years; 45% male), 249 participants completed the intervention (74% completion rate). Eligible participants were stratified by baseline Folstein Mini‐Mental State Examination (MMSE) score and age and randomised within each stratum to receive a daily supplement containing folic acid (400 μg), vitamin B_12_ (10 μg), vitamin B_6_ (10 mg) and riboflavin (10 mg) or placebo. Participants were invited to attend two 90‐minute appointments at the start and end of the trial. A researcher‐assisted questionnaire was administered to record biophysical, neuropsychiatric, cognitive and functional assessments, along with detailed information on nutritional intake, drug usage, medical and social history. Dietary intakes were assessed with the use of a 4‐days food diary combined with an FFQ, a method that was validated at Ulster University (United Kingdom) for the assessment of dietary intakes of folate, B_12_, B_6_ and riboflavin against each of their biomarkers (Hoey et al., 2007). Blood samples were collected and stored using standard operating procedures. Cognitive performance was assessed in detail at the start and end of the 2‐year trial using 3 cognitive tests: MMSE, the Frontal Assessment Battery (FAB), and the Repeatable Battery for the Assessment of Neuropsychological Status (RBANS). Anxiety and depression were also assessed using the Centre for Epidemiological Studies Depression scale and the Hospital Anxiety and Depression Scale. Functional brain activity was assessed at the end of the 2‐year intervention using magnetoencephalography in a subset of 48 participants with 24 from each of the placebo and treatment groups.

### Planned analysis of the EpiBrain project

Existing data and biobanked samples from the *BrainHOP* trial will be used in the *EpiBrain* project. DNA will be extracted and purified from blood samples of participants using a standard kit (Qiagen, UK). DNA will be treated with the chemical sodium bisulfite, which allows the detection of the methyl tags on inactivated genes. Assays will be run on the newly acquired state‐of‐the‐art Q48 pyrosequencing machine. To identify novel genes which may be inactivated/activated by changes in methylation in response to B vitamins, we will analyse epigenome‐wide changes using the EPIC array (Illumina) and confirm changes at the novel targets identified using locus‐specific pyroassays. We will then report on epigenomic changes in response to B vitamins in older adults in relation to cognitive performance and functional brain activity (as assessed using magnetoencephalography).

### The EpiBrain project aims and objectives

The overarching aim of the *EpiBrain* project is to investigate the nutrition–epigenome–brain relationship across the lifespan, focusing on methyl donor nutrients and their related epigenetic effects in relation to brain outcomes. This aim will be achieved through 3 distinct objectives, each addressed in the project's work packages (WPs):

1. To test if prenatal folic acid supplementation promotes cognitive development in the offspring with concomitant alterations in epigenetic signature (WP1).
2. To investigate epigenomic changes in response to B vitamin supplementation in relation to brain health and functional brain activity (using magnetoencephalography) in older adults (WP2).
3. To identify common epigenetic signatures related to cognition in both early and late life associated with folic acid (WP3).

## DISSEMINATION

The findings from the *EpiBrain* study will be disseminated through a wide range of communication channels, including outputs in scientific and health professional journals, communications via press releases, conference presentations, the *EpiBrain* website (www.epibrain‐folate.com), video summaries and social media (Twitter: @EpiBrain_JPI). Using these channels, and drawing on professional networks within our countries, the key findings will reach the European and international scientific community, policymakers and health authorities responsible for developing nutrition policies and strategies to promote health and prevent disease. We also aim to reach those tasked with monitoring nutritional status and generating dietary recommendations at a population level. In parallel, specific activities will target the general public, the food industry, relevant charity groups, and those with an interest in food, nutrition and health. Over the lifetime of the project and beyond it, the *EpiBrain* team will seek to build upon our collaboration and existing national and international networks, promote the project findings and develop further research and innovation.

## CONCLUSIONS

In summary, the *EpiBrain* project will build substantially on existing human cohorts and randomised trials from the United Kingdom, Canada and Spain to generate important new data relating to the nutrition–epigenome–brain relationship across the lifespan, with a particular focus on the one‐carbon nutrients – folate and the metabolically related B vitamins (vitamin B_12_, vitamin B_6_ and riboflavin). The outcomes will provide an improved understanding of the role of these B vitamins in the brain and the epigenetic mechanisms involved. Data from novel epigenetics analyses of our cohorts and intervention trials will be generated. The nutrition and epigenetics data generated from the project will also be linked with national data under the contrasting food fortification policies and micronutrient supplementation practices in place across our counties, thus ensuring that our outcomes have global reach and significance. Ultimately, our goal is to provide robust scientific evidence to support effective nutrition strategies to promote and sustain better brain health throughout life.

## AUTHOR CONTRIBUTIONS

Aoife Caffrey and Helene McNulty conceived the manuscript. Amy Tan prepared Figure 2. Aoife Caffrey, Yvonne Lamers, Michelle M. Murphy, Rachelle E. Irwin, Kristina Pentieva, Mary Ward, Amy Tan, Alejandra Rojas‐Gómez, L.A. Santos‐Calderón, J. Canals‐Sans, Brenda Leung, Rhonda Bell, Gerald Giesbrecht, Deborah Dewey, Catherine Field, Nicole Letourneau, Michael Kobor, Colum P. Walsh and Helene McNulty contributed to their respective work package descriptions, and reviewed, edited, and approved the final manuscript. Yvonne Lamers is the overall Project Coordinator for the *EpiBrain* project across the three participating countries.

## FUNDING INFORMATION

The *EpiBrain* Project was funded via an award from the European Joint Programming Initiative ‘A Healthy Diet for a Healthy Life’ (JPI HDHL) scheme for funding multilateral research projects under the ‘Nutrition and the Epigenome’ call. The funders within the participating countries were The Biotechnology and Biological Sciences Research Council (Grant BB/S020330/1, Ref. Prof. Helene McNulty, Ulster University, Northern Ireland, UK), The Spanish State Agency for Investigation (Grant PCI2018‐093098/AEI, Ref. Prof Michelle M Murphy, Universitat Rovira i Virgili, Catalonia, Spain) and The Canadian Institutes of Health Research (Grant 10R01093, Ref. Prof. Yvonne Lamers, University of British Columbia, Canada: Overall Project Coordinator).

## CONFLICT OF INTEREST STATEMENT

The authors have no conflicts of interest to declare.

## Data Availability

Data sharing not applicable to this article.
